# *Plasmodium falciparum *isolates from southern Ghana exhibit polymorphisms in the SERCA-type *PfATPase6 *though sensitive to artesunate *in vitro*

**DOI:** 10.1186/1475-2875-10-187

**Published:** 2011-07-11

**Authors:** Bethel Kwansa-Bentum, Irene Ayi, Takashi Suzuki, Joseph Otchere, Takashi Kumagai, William K Anyan, Joseph HN Osei, Hiroko Asahi, Michael F Ofori, Nobuaki Akao, Michael D Wilson, Daniel A Boakye, Nobuo Ohta

**Affiliations:** 1 Section of Environmental Parasitology, Department of International Health Development, Tokyo Medical and Dental University, 5-45 Yushima 1-chome, Bunkyo-ku, Tokyo 113-8519, Japan; 2 Parasitology Department, Noguchi Memorial Institute for Medical Research, P.O. Box LG 581, University of Ghana, Accra, Ghana; 3 Immunology Department, Noguchi Memorial Institute for Medical Research, P.O. Box LG 581, University of Ghana, Accra, Ghana; 4 Department of Parasitology, National Institute of Infectious Diseases, 23-1 Toyama 1-chome, Shinjuku-ku, Tokyo 162-8640, Japan

## Abstract

**Background:**

In 2005, Ghana replaced chloroquine with artemisinin-based combination therapy as the first-line treatment for uncomplicated malaria. The aim of this work was to determine for the first time, polymorphisms in the putative *pfATPase6 *and *pftctp*, *pfmdr1*, *pfcrt *genes in Ghanaian isolates, particularly at a time when there is no report on artemisinin resistance in malaria parasites from Ghana. The sensitivity of parasite isolates to anti-malaria drugs were also evaluated for a possible association with polymorphisms in these genes.

**Methods:**

The prevalence of point mutations in the above *Plasmodium falciparum *genes were assessed from filter-paper blood blot samples by DNA sequencing. *In vitro *drug sensitivity test was carried out on some of the blood samples from volunteers visiting hospitals/clinics in southern Ghana using a modified version of the standard WHO Mark III micro-test.

**Results:**

All successfully tested parasite isolates were sensitive to artesunate; while 19.4%, 29.0% and 51.6% were resistant to quinine, amodiaquine and chloroquine respectively. The geometric mean of IC_50 _value for artesunate was 0.73 nM (95% CI, 0.38-1.08), amodiaquine 30.69 nM (95% CI, 14.18-47.20) and chloroquine 58.73 nM (95% CI, 38.08-79.38). Twenty point mutations were observed in *pfATPase6 *gene, with no L263E and S769N. All mutations found were low in frequency, except D639G which was observed in about half of the isolates but was not associated with artesunate response (*p *= 0.42). The *pftctp *gene is highly conserved as no mutation was observed, while CVIET which is chloroquine-resistant genotype at codon 72-76 of the *pfcrt *gene was identified in about half of the isolates; this was consistent with chloroquine IC_50 _values (*p *= 0.001). Mutations were present in *pfmdr1 *gene but were not associated with artemisinin response (*p *= 1.00).

**Conclusion:**

The *pfATPase6 *gene is highly polymorphic with D639G appearing to be fixed in Ghanaian isolates. These may just be spontaneous mutations as all parasite isolates that were tested displayed satisfactory *in vitro *response to artesunate. However, there is no improvement in susceptibility of the parasites to chloroquine five years after its proscription.

## Background

Malaria remains a major public health concern in the world decades after Laveran discovered its causative agent in 1880. An estimated 247 million clinical cases occur every year across the globe, with about 881,000 deaths annually [1]. This quandary from malaria is partly due to the emergence and spread of *Plasmodium falciparum *parasites that are resistant to conventional anti-malaria drugs in almost all endemic countries [2,3]. Artemisinin derivatives, which are fast acting and kill all stages including young gametocytes of multi-drug resistant malaria parasites [4,5], have been recommended by the World Health Organization (WHO) in the form of combination therapy for the treatment of uncomplicated malaria to enhance its therapeutic efficacy as well as delay any possible resistance development in the human malaria parasite [6].

Studies in China, Thailand and Cambodia where oral artemisinin-based monotherapies have long been used, reported on reduced susceptibility to artemisinin derivatives in some malaria parasites [7-9]. Due to the fact that multi-drug resistant parasites originated from this area [10], diligent surveillance aimed at monitoring susceptibility of malaria parasites to artemisinin derivatives and the partner drugs in endemic areas is prudent. Pieces of evidence demonstrate that the sarco-endoplasmic reticulum calcium-dependent (SERCA) ATPase protein encoded by *pfATPase6 *and the translationally controlled tumour protein (TCTP) encoded by *pftctp *are involved in artemisinin activity [11,12].

Uhlemann *et al *[13] in their study observed that L263E replacement in *pfATPase6 *gene abolishes inhibition of artemisinins in *P. falciparum*. A subsequent study in French Guiana revealed that S769N mutation in *pfATPase6 *was associated with high artemether IC_50 _values [14]. In this same study, a combination of two mutations E431K and A623E in a clinical isolate from Senegal showed considerable increase in artemether IC_50 _[14], suggestive that these point mutations could be used in molecular monitoring of artemisinin derivatives to complement continuing *in vitro *surveillance. Until recently, it was thought that few mutations are present in the *pfATPase6 *gene as some studies have revealed polymorphisms in this putative gene even though their roles are not known [15,16]. Related studies have averred that while mutant haplotypes of *pfmdr1 *and *pfcrt *genes in some isolates conferred chloroquine resistance, they however increase parasite susceptibility to artemisinin derivatives [17,18].

In the year 2005, Ghana deployed artemisinin-based combination therapy (ACT) as the first-line treatment for malaria [19]; however, oral artemisinin-based monotherapies are also registered, and in an uncontrolled use by the general public [20]. In spite of this, there is no report of suspected artemisinin resistance in *P. falciparum *isolates from Ghana yet, and no work has been carried out on the *pfATPase6 *gene as a molecular marker in Ghana. In this study, the susceptibility of *P. falciparum *parasites to artesunate, amodiaquine, chloroquine and quinine was evaluated *in vitro *for Ghanaian isolates; point mutations in the *pfATPase6*, *pftctp*, and *pfmdr1 *genes were also examined, creating a picture in time before any report of resistance, and if they could be associated with differential responses to the anti-malaria drugs above. In addition, the putative *pfcrt *gene which is implicated in chloroquine resistance was evaluated for the status quo in the parasite isolates after five years of chloroquine proscription, as in the case of Malawi [21,22].

## Methods

### Study design and sites

This hospital/clinic based cross-sectional survey was carried out from August to October 2010. Seven communities in southern Ghana, Okyereko, Buduatta, Nyanyano, Apam, Kasoa, Achimota and Maamobi were selected based on the availability of a health facility, and proximity to Noguchi Memorial Institute for Medical Research (NMIMR), Accra-Ghana in order to reduce logistical constraints so that the blood samples will be cultured within eight hours after collection from participants. These areas were also purposively selected based on the fact that there is an already established acquaintance with the health centres and the community members for other on-going projects in NMIMR. Malaria transmission in Ghana is stable throughout the year with seasonal variation, while studies have shown that entomological inoculation rate in some of the study communities range from 0-20 infective bites/night [23,24].

Study participants were recruited from persons attending health centres in the above-mentioned communities. They included those who have been diagnosed with uncomplicated malaria on the basis of their clinical symptoms and by Rapid Diagnostic Test (RDT) kit (Malaria Ag Pf/Pan, Immunochromatographic assay, BIOLINE SD Rapid Test, STANDARD DIAGNOSTICS, INC, Haryana, India). All RDT-positive participants were given the appropriate treatment by the doctors in the respective health centres immediately after sample collection.

### Sample collection

A total of 146 strip filter-paper (ADVANTEC^®^) blood blots as well as thick and thin blood films were prepared from both *P. falciparum *antigen positive and negative RDT tested volunteers using finger-pricked blood samples. The filter-paper blood blots were air-dried and packaged into individual re-sealable plastic bags with silica gel desiccant and kept at 4°C until ready for genomic DNA extraction, while the slides were stained with Giemsa to adjust parasitaemia before culturing. Plain vacutainer tubes that were used for the blood collection were coated with 0.5 ml CPD-adenine buffer, an anticoagulant that can keep parasite isolates in good condition at room temperature for about eight hours [25]. Thirty-eight of the RDT-positives provided 3 ml of venous blood samples which were aseptically collected into the above tubes. These were transported to the laboratory within four hours of collection for drug sensitivity tests.

### Drug sensitivity tests

#### Malaria parasite culture medium

Hypoxanthine (SIGMA-Aldrich) solution was prepared by dissolving 54.4 mg in 40 ml distilled water with 0.6 ml of 2N-HCl. The basal medium consisted of RPMI 1640 with 2 mM glutamine and 25 mM HEPES (GIBCO-Invitrogen), with 25 μg/ml gentamicin solution (SIGMA-Aldrich). The culture medium comprised the above basal medium with 10% Daigo's GF21 (Wako Pure Chemical Industries, Japan), supplemented with 150 μM hypoxanthine and 5% normal human serum. This complete culture medium was termed HGRPMI [26].

#### Anti-malaria drugs tested

The anti-malaria drugs tested were appropriately reconstituted. 10 mM stock solution each of artesunate, amodiaquine dihydrochloride dihydrate (Sigma^®^) and chloroquine diphosphate salt (Sigma^®^) were initially prepared in 5 ml of phosphate buffered saline (PBS), while quinine (Sigma^®^) was dissolved with 70% ethanol. Final drug concentrations ranged from 0.16-160 nM for artesunate, 1.25-1280 nM for amodiaquine and chloroquine, and 10-10,240 nM for quinine. These were prepared using HGRPMI. All reagents and culture medium were filter-sterilized through 0.2 μm Acrodisc^® ^filter membrane after preparation.

#### Malaria parasite cultures

The sensitivity of Ghanaian isolates to the anti-malaria drugs was assessed by a modification of the standard WHO Mark III micro-test [27]. *In vitro *assays were carried out on samples with parasitaemia > 0.3% by microscopy, within eight hours after collection. The blood samples in CPD-adenine buffer were washed three times with basal medium (RPMI 1640), removing the supernatant and white blood cell interface after every wash at 1800 rpm for 6 min. Erythrocytes were re-suspended at 1:1 volume in HGRPMI. The starting parasitaemia was adjusted to 0.3-2.0% when required, by the addition of fresh uninfected erythrocytes. Each well of a 96-well tissue culture plate was filled with 90 μl drug solution of appropriate concentration plus 10 μl parasite suspension of about 5% haematocrit [28,29]. Control wells (with parasitized or uninfected erythrocytes) contained drug-free medium. Cultures of *P. falciparum *FCR3/FMG (ATCC Catalogue No. 30932, Gambia) was used as a reference strain. The plates were incubated at 37°C in a gas mixture of 90% N, 5% CO_2 _and 5% O_2 _for 25-30 hours.

### Growth inhibition assessment

At the end of the incubation period, suspended medium was removed while the blood within each well was used to make thick smears on a microscope glass slide. These were air-dried, stained with Giemsa and examined under microscope at ×100 magnification. The number of schizonts with three or more nuclei over a total of 200 asexual parasites was counted for each sample. All tests were done in duplicate; the drug concentration that inhibits schizogony by 50% (IC_50_) relative to the drug-free control samples of each *P. falciparum *isolate were estimated from dose-response curves by non-linear regression analysis using GraphPad Prism^® ^4.0 software package (GraphPad Software, San Diego, CA, USA). Confidence interval (CI) at 0.05 significance level was calculated by GraphPad QuickCalcs (Descriptive statistics and CI of mean/Confidence interval of SD) using the same software as above. Pearson's correlation coefficient was used to calculate the correlation between parasite responses to the anti-malaria drugs after entering data into Microsoft Office Excel.

### Genomic DNA extraction

Genomic DNA from each sample was obtained by boiling discs of the filter-paper blood blots in 150 μl of 50 mM NaOH with intermittent vortexing on a hot-plate (95°C 30'); 50 μl of 1 M Tris HCl was added to the recovered 50 μl supernatant and the resulting mixture directly used as template for gene amplification [30].

### Sequencing of *pfATPase6*, *pftctp*, *pfmdr1 *and *pfcrt *genes

Four specific overlapping oligonucleotide primer pairs (Table 1) were designed for genotyping significant portions of the coding sequence of *pfATPase6 *gene [GenBank:EF564342], while a pair of primer was sufficient for the relatively short *pftctp *gene [GenBank:DQ141561). Two pairs of primer were employed in sequencing portions of the *pfmdr1 *gene [GenBank:M29154.1] spanning codons 86, 184, 1034, 1042 and 1246; the first pair of primer targeted the first two codons whereas the second primer set were for the last three codons (Table 1). Two pairs of primer were also used in a nested PCR for sequencing portions of the *pfcrt *gene [GenBank:HQ287046] spanning codons 72-76 (Table 1). The 20 μl PCR mix contained 10 μl GoTaq^® ^Green Master Mix, primers at 0.5 μM final concentration, and 1 μl of DNA template. Cycling conditions for the various primer pairs are shown in Table 1. After gene amplification, the PCR products were run on 2% agarose gel electrophoresis to check for the correct bands; the products were directly sequenced with Big Dye terminator v3.1 Cycle Sequencing kit (Applied Biosystems, CA, USA), according to manufacturer protocol. Sequencing analyses were done in duplicates for all samples. The amplicon sequences were aligned with published data of the NCBI database by BLAST analysis.

**Table 1:** Oligonucleotide primers and PCR reaction conditions for SNPs detection in *pfATPase6*, *pftctp*, *pfmdr1 *and *pfcrt *genes

Gene Primer pair	Sequence 5' ⇒ 3'	PCR product size (bp)	PCR reaction conditions
***pfATPase6***			
atp6-1F	tcatctaccgctattgtatgtgg	777	94^o^C 5' followed by 40 cycles (94°C 15''; 55°C 30''; 72°C 40''); 72°C 5'
atp6-1R	attcctcttagcaccactcct		
atp6-2F	tcaccaaggggtatcaacaa	692	94°C 5' followed by 40 cycles (94°C 15''; 55°C 30''; 72°C 40''); 72°C 5'
atp6-2R	tggcataatctaattgctcttcc		
atp6-3F	atgtatagctgttgtaatcaacctaga	822	94°C 5' followed by 40 cycles (94°C 15''; 55°C 30''; 72°C 40'); 72°C 5'
atp6-3R	tcactatatggatcagcttcatca		
atp6-4F	ccagtacattgaatgaaaatg	605	94°C 5' followed by 40 cycles (94°C 15''; 55°C 30''; 72°C 40''); 72°C 5'
atp6-4R	acgtggtggatcaataatacct		

***pftctp***			
pftctp-1F	atgaaagtatttaaagacgtt	462	94°C 5' followed by 40 cycles (94°C 15'';
pftctp-1R	ttcttctcctttataataagaat		50°C 30''; 72°C 40''); 72°C 5'

***pfmdr1***			
mdr1-1F	tgaacaaaaagagtaccgctga	823	94°C 5' followed by 40 cycles (94°C 15''; 55°C 30''; 72°C 1'); 72°C 5'
mdr1-1R	ccataccaaaaaccgaatgc		
mdr1-2F	caagcggagtttttgcattt	1062	94°C 5' followed by 40 cycles (94°C 15''; 55°C 30''; 72°C 1'); 72°C 5'
mdr1-2R	ttctctgtttttgtccacctga		

***pfcrt***			
pfcrt-1'F	atggctcacgtttaggtgga		94°C 5' followed by 40 cycles (94°C 15''; 55°C 15''; 72°C 40''); 72°C 2'
pfcrt-2R	aaagcttcggtgtcgttc		
pfcrt-1F	tgtgctcatgtgtttaaactt		94°C 5' followed by 25 cycles (94°C 15''; 48°C 30''; 72°C 20''); 72°C 5'
pfcrt-2'R	ggaatagattctcttataaatcc	282	

The oligonucleotide primers were supplied by Greiner Bio-One Co., Ltd. Primer pairings of *pfATPase6 *gene, 1F & 1R, 2F & 2R, 3F & 3R, 4F & 4R were used resulting in the corresponding PCR product sizes. For the *pfcrt *primers, nested PCR was carried out with 1'F & 2R in the first reaction, followed by 1F & 2'R in the second reaction.

For any association between molecular and *in vitro *results, a threshold IC_50 _was set by halving the mean IC_50 _for each drug and then assigning each sample at either boundary of these values. The resulting data were then plotted onto 2 × 2 contingency tables and calculated for significance using two-tailed Fisher's exact test [28].

### Ethical clearance

Participants consented by signing an agreement form after having been informed of the study purpose, with entry and exit criteria explained. Permission to conduct this research and ethical clearance were obtained from the Scientific and Technical Committee, and the Institutional Review Board both of the Noguchi Memorial Institute for Medical Research, Ghana (Certified Protocol Number: 035/09-10). This study also received ethical approval from Tokyo Medical and Dental University, Japan (Certificate Number 955).

## Results

### *In vitro *drug sensitivity assessment

**Figure 1:**
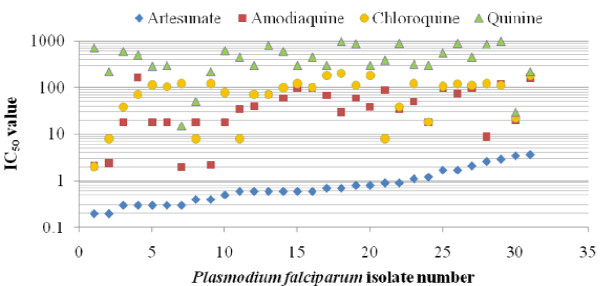
***In vitro *IC50 values for artesunate, amodiaquine, chloroquine and quinine for field isolates of *Plasmodium falciparum***. The isolates have been arranged in ascending order of artesunate IC50 values; thirty-eight isolates from patients diagnosed with uncomplicated malaria were used, out of which seven failed the tests due to poor growth/contamination.

Thirty-eight blood samples were phenotyped *in vitro *for their drug sensitivities, out of which 7 (18.4%) failed the test due to poor growth or contamination (Table 2 & Figure 1). No parasite isolate was resistant to artesunate, while 16/31 (51.6%) were resistant to chloroquine. The highest IC_50 _value for artesunate was 3.6 nM, with geometric means as follows: artesunate, 0.73 nM (95% CI, 0.38-1.08); amodiaquine, 30.69 nM (95% CI, 14.18-47.20); chloroquine, 58.73 nM (95% CI, 38.08-79.38); quinine, 355.37 nM (95% CI, 250.48-460.26) (Table 2). While Pearson's correlation coefficient between IC_50 _values of artesunate and amodiaquine was large (0.515), that of artesunate and chloroquine was medium, but were all statistically significant (Table 3). IC_50 _values for artesunate and chloroquine were 3.25 and 20.71 nM respectively for the reference strain, FCR3/FMG.

**Table 2:** *In vitro *drug sensitivity of *Plasmodium falciparum *isolates, *N = 31*

Anti- malaria drug	Number of isolates (%)		IC_50 _value (nM)		
	
	Sensitive	Resistant*	Lowest/Highest	Geometric mean (CI)	Standard deviation (CI)
Artesunate	31 (100)	0 (0)	0.2/3.6	0.73 (0.38-1.08)	0.95 (0.76-1.27)
Amodiaquine	22 (71.0)	9 (29.0)	2.0/162.0	30.69 (14.18-47.20)	45.00 (35.96-60.15)
Chloroquine	15 (48.4)	16 (51.6)	2.0/200.0	58.73 (38.08-79.38)	56.30 (44.99-75.25)
Quinine	25 (80.6)	6 (19.4)	15.0/990.0	355.37 (250.48-460.26)	285.95 (228.50-382.22)

*IC_50 _threshold for resistance are amodiaquine 60 nM, chloroquine 100 nM and quinine 800 nM. Studies on sensitivity cut-off point for artemisinin and its derivative is not conclusive yet but an IC_50 _of 20 nM is considered high [28,29]. Thirty-eight (38) samples were tested, out of which 31 (81.6%) were successfully phenotyped with the anti-malaria drugs. Test was successful when percentage of schizont to total parasite in drug-free control well was at least 10%. Confidence interval (CI) is at 0.05 significance level.

**Table 3:** Pearson's correlation coefficient (*r*) between IC_50 _values of the anti-malaria drugs

Anti-malaria drugs	Pearson's *r*	Interpretation	*p *value
artesunate *vs*. amodiaquine	0.515	Large	< 0.001
artesunate *vs*. chloroquine	0.366	Medium	< 0.001
artesunate *vs*. quinine	0.281	Small	< 0.001
amodiaquine *vs*. chloroquine	0.308	Medium	< 0.005
amodiaquine *vs*. quinine	0.188	Small	< 0.001
chloroquine *vs*. quinine	0.144	Small	< 0.001

Significance level was set at 0.05, while the range for interpretation of correlation coefficient is as follows; None, 0.0 - 0.09; Small, 0.1 - 0.3; Medium, 0.3 0.5; Large, 0.5 - 1.0.

### Prevalence of genetic polymorphisms in *pfATPase6 *and *pftctp *alleles

After amplifying portions of the genes, agarose gel electrophoresis of PCR products indicated that the bands were of the expected sizes. Out of the 146 filter paper blood blots that were collected for genotyping, 68 parasite isolates were successfully amplified for *pfATPase6 *and 52 for *pftctp *genes; these included 34 isolates (all the successfully tested isolates plus three unsuccessful ones) that were used for drug sensitivity test (phenotyping). A total of twenty point mutations were found in *pfATPase6 *gene (Table 4); analysis of this gene revealed a predominance of D639G in 34/68 (50%), E431K in 7/68 (10.3%), D443E in 5/68 (7.4%) and M813Q in 5/68 (7.4%) parasite isolates. Fisher's exact test revealed lack of association between D639G mutation and parasite response to artesunate (*p *= 0.423) (Table 5). All other mutations found were rare, while neither L263E nor S769N allele was observed. One sample harboured five mutations, another with four mutations while eight samples had three mutations each, and ten samples with two mutations each. Sequence comparisons of the *pftctp *gene revealed extreme conservation as all isolates exhibited the same identity.

**Table 4:** SNPs and their corresponding amino acid point mutations in *pfATPase6 *gene, *N = 68*

SNP	Point mutation	Frequency (%)	Artesunate IC_50 _(nM)
GGC	D639G	34 (50.0)	0.2 - 3.6^*^
AAA	E431K	7 (10.3)	0.6 - 3.6
GAA	D443E	5 (7.4)	0.6^*^
CAA	M813Q	5 (7.4)	0.6 - 0.9
CAT	Q622H	3 (4.4)	0.3 - 3.6
GTA	L402V	2 (2.9)	0.3 - 2.6
TTA	F414L	2 (2.9)	0.4 - 0.8
AAT	D419N	2 (2.9)	0.3
TAT	C356Y	1 (1.5)	1.4
AAA	R377K	1 (1.5)	NA
AAA	E384K	1 (1.5)	NA
AAA	T403K	1 (1.5)	0.2
GAA	D405E	1 (1.5)	1.7
ACT	A425T	1 (1.5)	NA
AAA	E432K	1 (1.5)	NA
TCT	A630S	1 (1.5)	NA
GGT	C645G	1 (1.5)	0.5
GGA	E696G	1 (1.5)	NA
AAA	E710K	1 (1.5)	0.4
GGT	D734G	1 (1.5)	NA

*Some isolates of the corresponding nucleotide were not phenotyped, just as NA (not applicable) represents isolates that were not phenotyped.

**Table 5:** Fisher's exact test of association between polymorphisms and *in vitro *drug response, *N = 31*

Polymorphism *vs*. drug	*p *value
*pfcrt *(K76T) *vs*. chloroquine	0.001
*pfATPase6 *(D639G) *vs*. artesunate	0.423
*pfcrt *(K76T) *vs*. artesunate	1.000
*pfmdr1 *(N86Y) *vs*. artesunate	1.000
*pfmdr1 *(Y184F) *vs*. artesunate	1.000
*pfmdr1 *(N86Y) *vs*. chloroquine	1.000
*pfmdr1 *(Y184F) *vs*. chloroquine	0.104
*pfmdr1 *(N86Y) *vs*. amodiaquine	1.000
*pfmdr1 *(Y184F) *vs*. amodiaquine	0.253
*pfmdr1 *(N86Y) *vs*. quinine	1.000
*pfmdr1 *(Y184F) *vs*. quinine	1.000
*pfmdr1 *(double mutant) *vs*. artesunate	0.577
*pfmdr1 *(double mutant) *vs*. chloroquine	1.000
*pfmdr1 *(double mutant) *vs*. amodiaquine	0.630
*pfmdr1 *(double mutant) *vs*. quinine	1.000

### Prevalence of genetic polymorphisms in *pfmdr1 *and *pfcrt *alleles

**Figure 2:**
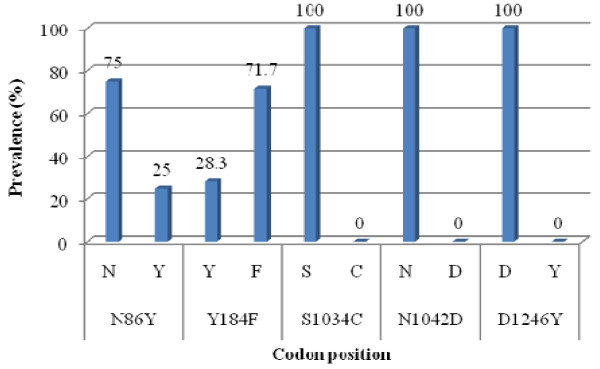
**Prevalence of genetic polymorphisms in the *pfmdr1 *gene, *N = 60***. All N86Y mutants possessed the TAT nucleotides while all Y184F harboured the TTT nucleotides. Twenty-two (36.7%) samples were double mutants and 30 (50%) samples had the single mutant Y184F.

Sixty parasite isolates were successfully amplified for both *pfmdr1 *and *pfcrt *genes, with 34 isolates phenotyped. Fifteen (25%) isolates harboured the N86Y mutation (TAT nucleotides) while 43/60 (71.7%) with Y184F mutation (TTT nucleotides) in the *pfmdr1 *gene (Figure 2). Twenty-two (36.7%) samples were double mutants and 30/60 (50%) had the single mutant Y184F. None of these mutants was found to be associated with drug response which was supported by Fisher's exact test (Table 5). A total of 29/60 (48.3%) CVIET and 31/60 (51.7%) CVMNK, which are chloroquine-resistant and chloroquine-sensitive genotypes respectively were identified at codons 72-76 of *pfcrt *gene. This was consistent with the *in vitro *culture but there was no particular association with artesunate response. This observation was corroborated by Fisher's exact test (Table 5). There was no observed correlation between *pfATPase6 *D638G and *pfcrt *K76T with Pearson's correlation coefficient value of -0.178 and Fisher's exact test of 0.738 at *p *= 0.05.

## Discussion

The use of oral artemisinin-based monotherapies alongside ACT is a common practice in Ghana [20], a situation that if not monitored and controlled threatens the efficacy of the latter. In the latest World Malaria Report, WHO admonishes all endemic countries to be more vigilant in monitoring anti-malaria drug efficacy for early detection of artemisinin resistance [31]. Alam *et al *[32] reported on *pfcrt*, *pfmdr1*, *dhps *and *dhfr *mutations associated with chloroquine and sulphadoxine-pyrimethamine resistance and the microsatellite loci flanking these genes in *P. falciparum *isolates from Ghana. There is no report on *pfATPase6 *genotype from this part of the world. In this study, we determined the prevalence of point mutations in *pfATPase6*, *pftctp*, *pfmdr1*, *pfcrt *genes and partly evaluated the susceptibility of *P. falciparum *parasites to artesunate, amodiaquine, chloroquine and quinine *in vitro*.

Studies have shown that parasites from the African continent appear to be more susceptible to artemisinin derivatives [28,33,34]. In this study, all field isolates of *P. falciparum *that were tested exhibited satisfactory *in vitro *response to artesunate, while more than half were resistant to chloroquine. What is more important is the presence of parasite isolates that are resistant to amodiaquine in the phase of continual use of oral artemisinin-based monotherapies. Ferreira *et al *[28] in their study observed a strong correlation between artesunate and amodiaquine; its significance in terms of factors that lead to sensitivity must be studied since this observation was replicated in our study. Oduro *et al *[35] reported on reduced cure rate of amodiaquine in northern Ghana. While it has been observed that persons infected with artemisinin resistant parasites could be cured by ACT, cure rate largely depended on efficacy of the non-artemisinin component of the combination [31]. This calls for speedy action on withdrawal of oral artemisinin-based monotherapies from the Ghanaian market. For effective use of these drugs in the control of malaria, there is the need for generation of molecular epidemiological data, ideally before the introduction of such drugs since mutations could be selected by previous drug pressure. Evaluation of polymorphisms in potential candidate genes of drug resistance is of utmost importance to establishing their role in modulating drug response, and to predicting epidemiological dynamics of resistance.

The limitation to this study was that polymorphisms in the *pfATPase6 *gene was investigated after five years of drug (artemisinin and its derivatives) pressure in Ghana; nonetheless, this study presents a picture in time since there is no report of artemisinin resistance in Ghanaian isolates. This study therefore describes for the first time, polymorphisms in *pfATPase6 *gene in such isolates. By using PCR sequencing assay, neither L263E nor S769N point mutation was observed; and this is consistent with the *in vitro *micro-test results. A total of twenty point mutations were observed in this gene, confirming its molecular diversity than previously demonstrated [15,36,37]; these however, may be spontaneous mutations since their role in artemisinin sensitivity is not yet defined. D639G mutation was observed in about half of the parasite isolates, and thus appears to be fixed in the parasite population. This mutant is supposed to be positioned in the cytosolic region located between transmembrane domains 5 and 6, albeit its biological significance is difficult to forecast in the absence of functional analysis [29].

Even though studies have shown the involvement of the malaria parasite *pftctp *gene in artemisinin activity, our present result confirms its extreme conservation, and thus not a strong candidate for resistance to artemisinin and its derivatives due to the fact that it is not under major selective drug pressure [14,29]. Studies have suggested that mutant haplotypes of *pfmdr1 *and *pfcrt *genes in some isolates conferred chloroquine resistance [38,39], but however increase parasite susceptibility to artemisinin [17,18]. Our study revealed the presence of two mutations in the *pfmdr1 *gene and K76T allele in the *pfcrt *gene; but they had no part in the modulation of artesunate response as the lowest and highest artesunate IC_50 _values corresponded to parasite isolates with *pfcrt *K76T. This was corroborated by Fisher's exact test. Afonso *et al *[40] in their study observed that malaria parasites, such as *Plasmodium chabaudi*, can develop stable resistance to artemisinin but lack mutations in candidate genes including *atp6*, *tctp*, *mdr1 *and *cg10*.

More than half of the parasite isolates were resistant to chloroquine after five years of its proscription in Ghana, which is a reflection of our molecular results but at variance to the Malawian situation where sensitive isolates were recovered after stopping the use of chloroquine [21,22]. The K76T allele was strongly associated with this behaviour, which was confirmed by Fisher's exact test. This may be due to the persistent use of chloroquine in some parts of Ghana [20].

## Conclusion

*Plasmodium falciparum *isolates from southern Ghana showed no improvement in susceptibility to chloroquine, but they exhibited satisfactory *in vitro *response to artesunate. No L263E and S769N mutations in the SERCA-type *PfATPase6 *were observed, albeit evidence is clear on the greater molecular diversity of *PfATPase6 *than previously demonstrated as twenty mutations were detected with D639G appearing to be fixed in this parasite population. Whereas these mutations may be spontaneous and as such without any particular role in the parasites response to artemisinin, continual monitoring of *P. falciparum *susceptibility to artemisinin is welcome.

## Competing interests

The authors declare that they have no competing interests.

## Authors' contributions

BKB and NO conceived the idea, while BKB, IA, TS, JO, WKA and JHNO performed the field work. BKB, IA, TS, JO, JHNO, HA, MFO and NA did the *in vitro *drug assessment test while BKB and TK did the molecular work. NO, MDW and DAB were involved in all stages. BKB wrote the manuscript, which was read and approved by all authors.
